# Is the term substitution relevant to Pharmacognosy and/ or vegetable crude drug industry?

**DOI:** 10.4103/0974-8490.72333

**Published:** 2010

**Authors:** A. B. D. Selvam

**Affiliations:** *Pharmacognosy Section, Botanical Survey of India, Howrah - 711 103, India*

**Keywords:** Substitution, alternative, alternative source, pharmacognosy, crude drug industry

## Abstract

Since each and every drug plant has its own characteristic features, in terms of its botanical characters, chemical composition and therapeutic properties, considering the highly potential drug plants as genuine plant and less potential (allied or non-allied) drug plants as substitutes is unjustifiable. Moreover, the term Substitution is being used for a couple of centuries in a wrong sense in pharmacognostic studies or in the vegetable crude drug industry. Therefore, the term ’Substitution’ has to be replaced by the relevant and appropriate term, ’Alternative’ or ’Alternative source’.

## INTRODUCTION

Pharmacognosy is the scientific study of crude drugs from four different natural sources, namely, plants, animals, minerals and metals. More than 90% of the crude drugs are derived from plant sources, while the remaining are from animal, metal and mineral sources.

The vegetable crude drugs are studied pharmacognostically using five customary parameters, which are the botanical, zoological (organoleptic), physical, chemical, and biological (pharmacological) parameters, aimed at disseminating the unique features of vegetable crude drugs in three different phases, namely, identification, isolation of active principles / compounds, and screening for biological activities.

The pharmacognosists often come across two familiar practices, namely ‘adulteration’ and ‘substitution,’ which are prevalent in trade nowadays. Adulteration, in a broad and legal sense, is the debasement of any article, which involves conditions such as inferiority, spoilage, deterioration, admixture, sophistication, and substitution. Adulterating the crude drugs by any of the said conditions is considered undesirable in the crude drug industry.

Adulteration, therefore, may be referred to as a fraudulent activity or malpractice and is akin to cheating, whereas, substitution is not so. It is apparent from the above-mentioned definition that the term ′substitution′ has long been used in a wrong sense as one of the conditions of ‘adulteration’, which is a separate entity.

The word ‘substitution’ means ‘an article or a person is put in place of another article or a person who is no longer available or put in exchange for’, whereas, pharmacognostically, it is defined as ‘an entirely different article that is used or sold in place of the required or requested article’. Cottonseed oil sold as Olive oil and American saffron sold as Spanish saffron are examples of substitution.[1]

## EXPLANATIONS

The term ‘substitution’ should be specifically used when a crude drug that was once available in the crude drug market is presently unavailable, and hence, another crude drug (either allied or non-allied) is used in its place, which possesses more or less similar properties, that is, the available crude drug is used in place of the unavailable crude drug. An example given herewith will elaborate how the term substitution is wrongly used in the Indian crude drug industry or in pharmacognostic studies. The root, root bark, and stem of the drug plant *Berberis aristata* DC (Berberidaceae) and the root, stem, and stem bark of the drug plant *Coscinium fenestratum* (Gaertn.) Colebr. (Menispermaceae) have been in trade / in use in the Indian crude drug market / industry for a very long time. However, the former drug plant is considered as genuine and the latter as a substitute.[2]

The root bark, wood, and an extract made from the root bark (*Rasaut*) of *Berberis aristata* are considered as alterative and deobstruent and are useful in skin diseases, menorrhagia, diarrhoea, jaundice, and affections of eyes. The decoction of the root bark of this plant is used in malarial fever. The dried stem is considered as a bitter tonic and is useful in treating intermittent fevers. Further, the root bark of this plant contains an alkaloid called *berberine* and the roots and the stem yield a yellow dye.[3,4] Even as the root of *Coscinium fenestratum* is considered to be a bitter tonic, stomachic and antiseptic, and is useful in treating dysentery, dressing wounds, and ulcers, the wood of this plant is also considered as a bitter tonic. The decoction of the stem is useful in snake-bites and the decoction of stem bark is useful in intermittent fevers. This plant contains an alkaloid *berberine*. Furthermore, the stem yields a yellow dye, which is useful in treating dyspepsia and is also used as a febrifuge and for dressing wounds and ulcers.[3,4]

Although both the above-mentioned drug plants are referred to as ‘*Daru haridra*’ in Sanskrit and share some common features such as possessing an alkaloid ‘*Berberine*’ and also yielding a yellow dye, the therapeutic properties and their uses are almost dissimilar as detailed earlier. Therefore, considering one drug plant as genuine and the other as a substitute is strongly questionable? In the same way, almost all the drug plants that are treated either as genuine or substitute in pharmacognostic studies or in the vegetable crude drug industry differ from one another in a number of features.

In addition, it should be noted that the drug plant *Coscinium fenestratum*, which is considered as a substitute in the pharmacognostic study or in the vegetable crude drug industry has been enlisted by the Government of India, in 1998, in the Negative list of Exports. Considering the rarity of this plant species in the wild, it needs to be conserved for posterity. Furthermore, the trade / export of the negative listed plants have been banned from wild collections.

Let us take a live example from one of the Pharmacognosy-related disciplines, to discuss about the right usage of the term substitution. We use a variety of medical systems across the globe. Every system is unique in its methodology and treatment, despite the fact that all the systems are attempting or aiming at a single destination, that is, curing human ailments (diseases / disorders). However, only the Allopathic system (English or modern medicine) is recognized and used as a main system of medicine throughout the world. Even though the remaining medical systems are more or less as competent as English medicine, they are still considered as alternative systems of medicine (many of them are traditional systems of medicine) and not as substitute systems to English medicine. Following the aforesaid example, the drug plants that are considered as ‘substitute’ should be treated / considered as ‘alternative’ or ‘alternative source’.

## CONCLUSION

Since every drug plant has unique features in terms of its botanical characters, chemical composition and therapeutic properties, considering the highly potential drug plants as genuine and less potential (allied or non-allied) drug plants as substitutes is unjustifiable. It is definitely a wrong concept and should be corrected forthwith.

In the light of the above-mentioned facts, it is concluded that the term ‘substitution’ is an irrelevant term, which has been in use in pharmacognostic studies or in the vegetable crude drug industry for a couple of centuries, and must be replaced by the relevant and appropriate term, ‘alternative’ or ‘alternative source’. It is further suggested that before replacing the term ‘Substitution’ with ‘Alternative’, it could be discussed among the pharmacognosists, to arrive at a suitable conclusion in this regard.
